# H3S28 phosphorylation is a hallmark of the transcriptional response to cellular stress

**DOI:** 10.1101/gr.176255.114

**Published:** 2014-11

**Authors:** Anna Sawicka, Dominik Hartl, Malgorzata Goiser, Oliver Pusch, Roman R. Stocsits, Ido M. Tamir, Karl Mechtler, Christian Seiser

**Affiliations:** 1 Department of Medical Biochemistry, Max F. Perutz Laboratories, Medical University of Vienna, Vienna Biocenter, 1030 Vienna, Austria;; 2 Research Institute of Molecular Pathology, 1030 Vienna, Austria;; 3 Center for Anatomy and Cell Biology, Medical University of Vienna, 1090 Vienna, Austria;; 4 Campus Science Support Facilities GmbH, 1030 Vienna, Austria;; 5 Protein Chemistry Facility, IMBA Institute of Molecular Biotechnology of the Austrian Academy of Sciences, 1030 Vienna, Austria

## Abstract

The selectivity of transcriptional responses to extracellular cues is reflected by the deposition of stimulus-specific chromatin marks. Although histone H3 phosphorylation is a target of numerous signaling pathways, its role in transcriptional regulation remains poorly understood. Here, for the first time, we report a genome-wide analysis of H3S28 phosphorylation in a mammalian system in the context of stress signaling. We found that this mark targets as many as 50% of all stress-induced genes, underlining its importance in signal-induced transcription. By combining ChIP-seq, RNA-seq, and mass spectrometry we identified the factors involved in the biological interpretation of this histone modification. We found that MSK1/2-mediated phosphorylation of H3S28 at stress-responsive promoters contributes to the dissociation of HDAC corepressor complexes and thereby to enhanced local histone acetylation and subsequent transcriptional activation of stress-induced genes. Our data reveal a novel function of the H3S28ph mark in the activation of mammalian genes in response to MAP kinase pathway activation.

Inducible transcription programs converge on the activation of sequence-specific transcription factors that recruit histone-modifying enzymes, which enables the deposition of stimulus-specific chromatin modifications at signal-responsive gene regulatory elements (Smith and Shilatifard 2010; Johnson and Dent 2013). One example of such a signal-inducible chromatin mark that couples signal transduction to gene regulation is the phosphorylation of histone H3. Since histone H3 was identified as a potential substrate for a signaling kinase (Halegoua and Patrick 1980), an increasing body of evidence suggests an important role for histone H3 phosphorylation in the regulation of signal-inducible transcription in mammals (Banerjee and Chakravarti 2011; Sawicka and Seiser 2012). Specifically, rapid and transient phosphorylation of histone H3 at S10 and S28 by MSK1 and MSK2 downstream from the ERK and p38 MAP kinase pathways, termed the nucleosomal response, has been implicated in the transcriptional activation of immediate early (IE) genes (Mahadevan et al. 1991; Cheung et al. 2000a; Clayton and Mahadevan 2003). Several studies have provided insight into the mechanistic aspects of histone H3 phosphorylation in transcriptional regulation; however, genome-wide approaches in mammalian inducible transcription systems have not yet been reported. Importantly, the evidence that has accumulated from analysis of single genes indicates that histone H3 phosphorylation influences the association of factors involved in gene expression control. In particular, H3S10 phosphorylation in combination with acetylation of H3K9 or H3K14 residues was shown to recruit the 14-3-3 proteins and thereby promote the activation of *Hdac1, Cdkn1a,* several IE genes, as well as *VL30* transposable elements (Cheung et al. 2000b; Thomson et al. 2001; Clayton and Mahadevan 2003; Mahadevan et al. 2004; Winter et al. 2008b; Brunmeir et al. 2010; Drobic et al. 2010; Simboeck et al. 2010). In the case of the *Fosl1* enhancer, 14-3-3 recruitment to phosphorylated H3S10 initiates a cascade of events leading to the release of RNA Polymerase II (RNAPII) from the promoter–proximal paused state (Zippo et al. 2009). In contrast to H3S10ph, the impact of H3S28 phosphorylation on stimulus-induced transcriptional regulation is much less studied (Sawicka and Seiser 2012). Interestingly, phosphorylation of H3S28 was demonstrated to counteract Polycomb silencing by facilitating dissociation of Polycomb repressive complexes in response to external signaling (Gehani et al. 2010; Lau and Cheung 2011). Despite insights from several biological systems, the mechanisms by which signal-inducible histone H3 phosphorylation impacts the transcription are still not fully understood.

Here we provide a comprehensive analysis of transcriptional response to stress, and for the first time report the genome-wide distribution and functional role of H3S28ph in a mammalian system. Using a combination of ChIP-seq, RNA-seq, and mass spectrometry approaches, we identified genomic targets of stress-induced H3S28ph and factors involved in the biological interpretation of this histone modification. This systematic approach enabled us to identify a novel mechanism of H3S28ph-mediated modulation of histone acetylation levels. Our data strongly support a model in which stress-induced phosphorylation at H3S28 reduces the association of histone deacetylase (HDAC)-containing complexes, thereby promoting a local increase in histone acetylation.

## Results

### Genome-wide distribution of the H3S28ph mark in stress-induced mouse 3T3 fibroblasts

**Figure 1.**
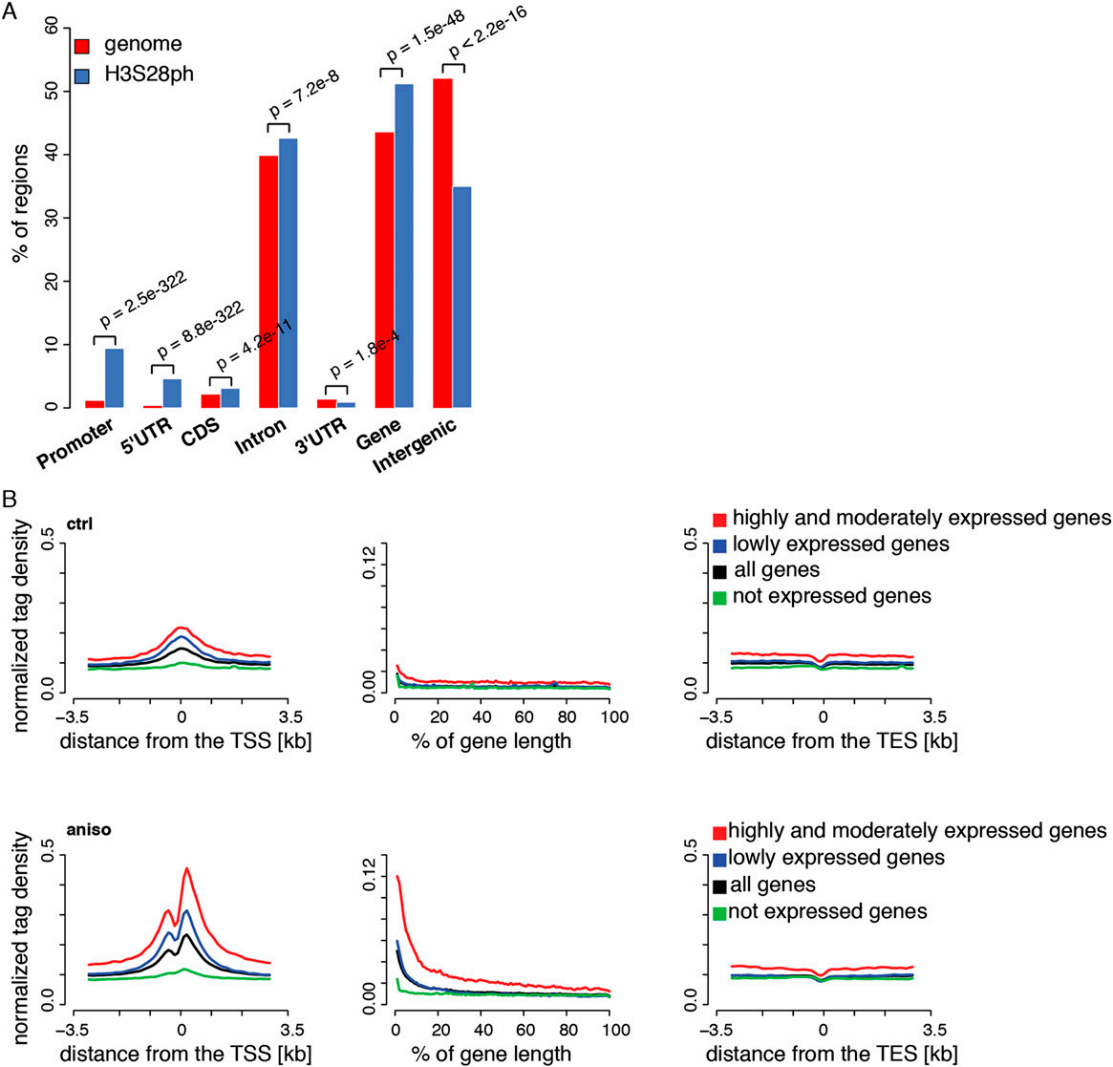
Stress-induced H3S28ph targets active promoters. (*A*) Enrichment of H3S28ph ChIP-seq regions over six gene features compared with the genome background. “Promoter” is defined as the region located within 1 kb upstream of the annotated transcription start site (TSS). “CDS” refers to the coding sequence. “Gene” consists of 5′UTR, coding exons, introns, and 3′UTR. “Intergenic” refers to all regions located outside of genes extended by 1 kb from both gene ends. The significance of the observed enrichment was determined by a one-sided binomial test. (*B*) Average density plots of normalized H3S28ph ChIP-seq tags centered at transcription start sites (TSS, *left* panel), transcription end sites (TES, *right* panel) for a control (ctrl), and stress-induced state (aniso). (*Middle* panel) The metagene profile of H3S28ph ChIP-seq densities. The genes are grouped according to their expression level: highly and moderately expressed (in red; ctrl: 3429 genes, aniso: 3181 genes), lowly expressed (in blue; ctrl: 7271 genes, aniso: 7369 genes), and not expressed (in green; ctrl: 10,908 genes, aniso: 11,059 genes). The average profile derived from all genes is depicted in black. The classification of the genes is based on mRNA-seq data (see Supplemental Methods and Supplemental Fig. 4B).

Numerous studies have addressed the role of histone H3 phosphorylation in the transcriptional regulation of specific mammalian genes in response to extracellular signals (Cheung et al. 2000b; Saccani et al. 2002; Yamamoto et al. 2003). However, since these approaches were limited to individual loci chosen a priori, the genome-wide localization, as well as the number of genomic locations targeted by this modification, remain largely unknown in the mammalian system. In order to study the phosphorylation of H3S28 in signal-induced transcription, we took advantage of the well-characterized system of serum-deprived mouse Swiss 3T3 fibroblasts that are arrested in the G_0_ phase of the cell cycle. In these cells, triggering of the p38 MAP kinase pathway with the stress inducer anisomycin enabled us to study the phosphorylation of H3S28 in the absence of excessive mitotic histone H3 phosphorylation found at condensed chromosomes (Spite et al. 2007). To explore the genome-wide distribution of the H3S28ph mark in quiescent and stress-stimulated cells, we performed chromatin immunoprecipitation analysis coupled with massively parallel sequencing (ChIP-seq) in anisomycin-treated and untreated serum-deprived fibroblasts. Specificity of the H3S28ph antibody was tested using a set of differentially modified histone H3 peptides. This analysis has proven its high specificity toward H3S28ph, insensitivity to modifications occurring at the neighboring K27 residue, as well as a lack of cross-reaction with histone peptides bearing H3S10ph, which is embedded in the same ARKS amino acid motif as the H3S28 residue (Supplemental Fig. 1). ChIP-seq analysis of H3S28 phosphorylation was performed with two biological replicates that agreed well (Spearman’s correlation coefficient 0.75; Supplemental Fig. 2A; Supplemental Table 1). We identified 2480 genes associated with the H3S28ph mark in stress-induced cells (FDR ≤ 0.05 and fold enrichment ≥ 5; Supplemental Tables 2, 3). Importantly, no H3S28ph-marked genes were identified in untreated cells using these thresholds, demonstrating a lack of background phosphorylation in our system and, therefore, its suitability for studying signal-induced histone H3 phosphorylation. The H3S28ph mark was enriched at promoters and 5′ untranslated regions (UTRs) (Fig. 1A), with 53% of H3S28ph-marked regions overlapping with CpG islands. Gene ontology (GO) analysis of H3S28ph targets revealed a highly significant enrichment for genes with molecular functions in signaling, transcriptional control, and nucleoside/nucleotide binding, cellular metabolism regulation, intracellular transport, and cell death (Supplemental Fig. 3; Supplemental Table 4). This is in agreement with a well-established role of the p38 MAPK pathway in regulating these processes (Deacon et al. 2003; Khurana and Dey 2003; Gehart et al. 2010). In addition, a high enrichment for genes involved in development and morphogenesis raises the possibility that a fraction of H3S28ph-marked genes is regulated by Polycomb, consistent with recent findings that H3S28ph mediates the dissociation of Polycomb repressive complexes (PRCs) (Gehani et al. 2010; Lau and Cheung 2011).

### Stress-induced H3S28ph positively correlates with active transcription

To gain insight into the localization of H3S28ph in the context of chromatin architecture and gene expression on a global scale, we performed systematic analysis of the transcriptional response to stress in serum-deprived mouse Swiss 3T3 fibroblasts treated with anisomycin for 1 h and untreated serum-deprived cells as a control. To this end, we performed gene expression profiling using full-length mRNA-seq as well as ChIP-seq of two histone modifications characteristic for chromatin in the transcriptionally permissive state, H3K9ac and H3K4me3, together with initiation-competent and elongating forms of RNA Pol II (phosphorylated at S5 and S2 of its carboxy-terminal domain, respectively) (Supplemental Tables 1*–*3). Biological replicates of all the experiments were highly correlated (Supplemental Figs. 2B–E, 4A). To examine the distribution of H3S28ph at different functional regions, we generated normalized tag density profiles for regions surrounding the transcription start sites (TSSs) and transcription end sites (TESs), as well as entire gene bodies for groups of genes divided according to their expression levels (see Fig. 1B; Supplemental Fig. 4B for details). We found that stress-induced H3S28ph marks preferentially accumulate around the TSS of expressed genes and positively correlate with gene activity (Spearman’s rank correlation coefficient 0.68; Supplemental Fig. 5). Notably, 2034 out of 2480 H3S28ph-marked genes (87%) showed detectable levels of expression in untreated cells (log_2_ RPKM mRNA-seq in ctrl ≥ 0). Given the correlation between H3S28ph and gene expression levels, we next compared the H3S28ph density around TSSs to the densities of H3K9ac and H3K4me3 marks and RNAPII. As expected, H3S28ph coincided with the presence of H3K9ac, H3K4me3, and both RNAPII isoforms (RNAPIIS5ph and RNAPIIS2ph) (Supplemental Fig. 6). To test whether stress-induced H3S28ph requires active transcription, we pretreated the cells with general transcriptional inhibitors actinomycin D or triptolide (Titov et al. 2011) prior to stress stimulation. Global phosphorylation of H3S28 as well as specific association of the H3S28ph mark with the target genes *Dusp1*, *Mafk*, *Traf1*, and *Nfil3* (Supplemental Fig. 7) were largely unaffected by these inhibitors, further demonstrating that the H3S28 phosphorylation patterns we observed were unlikely to result from active transcription.

### Stress-induced genes are transcriptionally active under basal conditions

**Figure 2.**
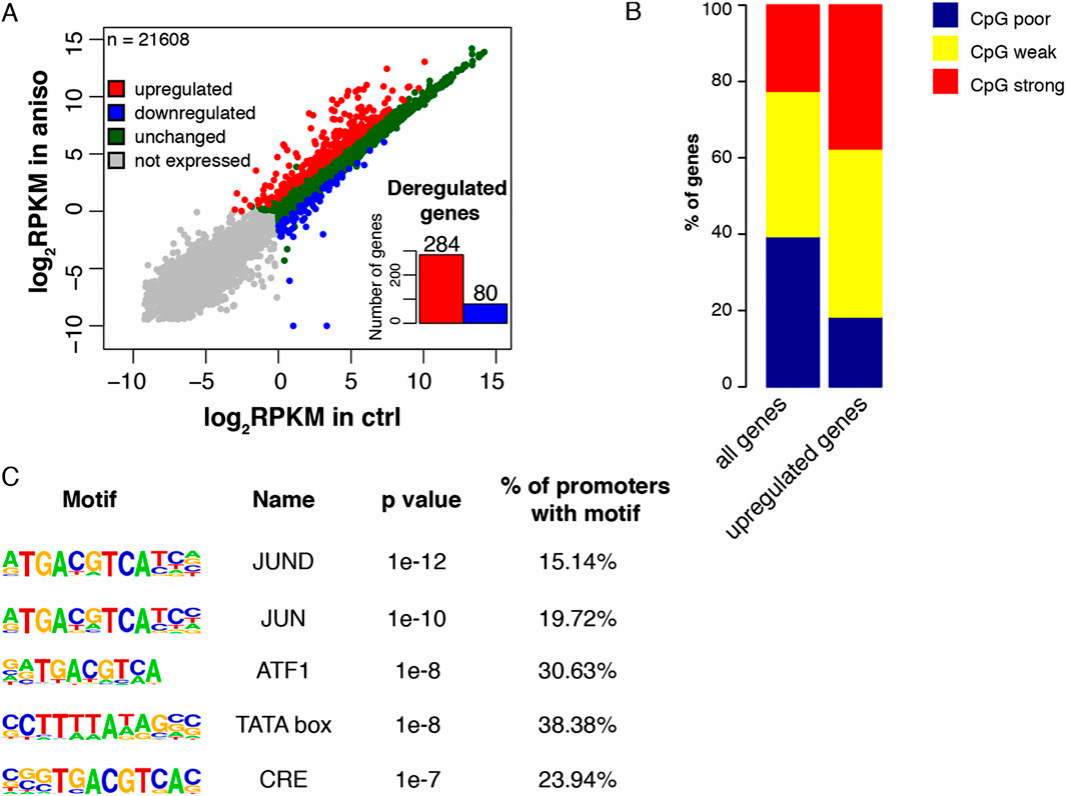
Stress signaling via p38/MAPK mainly targets the regulators of signal transduction characterized by the high CG content of their promoters. (*A*) Changes in mRNA abundance of 21,608 RefSeq genes upon stress stimulation determined by full-length mRNA-seq. (*B*) Histogram showing the distribution of promoter classes according to CpG content. Weak and strong CpG promoters are enriched in the group of up-regulated genes (*P*-value = 1.052 × 10^−14^, χ^2^ test). (*C*) Transcription-factor motifs enriched in promoter regions (located between 400 bp upstream of and 100 bp downstream from the TSS) of up-regulated genes.

In order to determine the changes in transcript abundance upon anisomycin treatment, we performed a differential expression analysis of the full-length mRNA-seq data. We identified 364 differentially expressed genes, 284 of which showed an increase in their mRNA levels upon stress induction (fold change ≥ 2, *P*-value < 0.01) (Fig. 2A). GO analysis of the up-regulated genes revealed an enrichment in regulatory components of the signaling pathways as well as the factors involved in transcriptional regulation of metabolic and developmental processes (Supplemental Fig. 8; Supplemental Table 5). This is consistent with the role of primary response genes in the propagation of the signal inside the cell to establish a proper cellular response to environmental cues (Herschman 1991). Importantly, 260 out of 284 up-regulated genes (92%) showed detectable levels of expression in untreated cells (log_2_ RPKM mRNA-seq ≥ 0), suggesting that the IE response to stress first acts to amplify the transcription of already expressed genes rather than to establish an active state of silent ones, similar to other inducible transcription programs (Hargreaves et al. 2009; Escoubet-Lozach et al. 2011). It has been demonstrated that primary response genes are characterized by high CpG content of their promoters (Hargreaves et al. 2009; Ramirez-Carrozzi et al. 2009). In order to determine whether this is also a feature of stress-induced genes, we analyzed the CpG content of genes up-regulated upon anisomycin treatment according to the previously established criteria (Mohn et al. 2008). Indeed, we found an enrichment for weak and strong CpG promoters (*P*-value = 1.052 × 10^−14^, χ^2^ test) in the set of stress-induced genes (Fig. 2B). Moreover, analysis of promoter sequences (400 bp upstream of and 100 bp downstream from the TSS) of the up-regulated genes identified binding sites for factors regulated by p38 kinase activity such as JUN, JUND, ATF1, and CREB1 (Fig. 2C; Tan et al. 1996; Gao et al. 2013). In addition, we found an enrichment for the TATA box motif in promoter sequences of the up-regulated genes (Fig. 2C), in agreement with the well-established role of this motif in the regulation of signal-inducible transcription (Basehoar et al. 2004; Yang et al. 2007).

### H3S28ph marks a significant fraction of stress-induced genes

**Figure 3.**
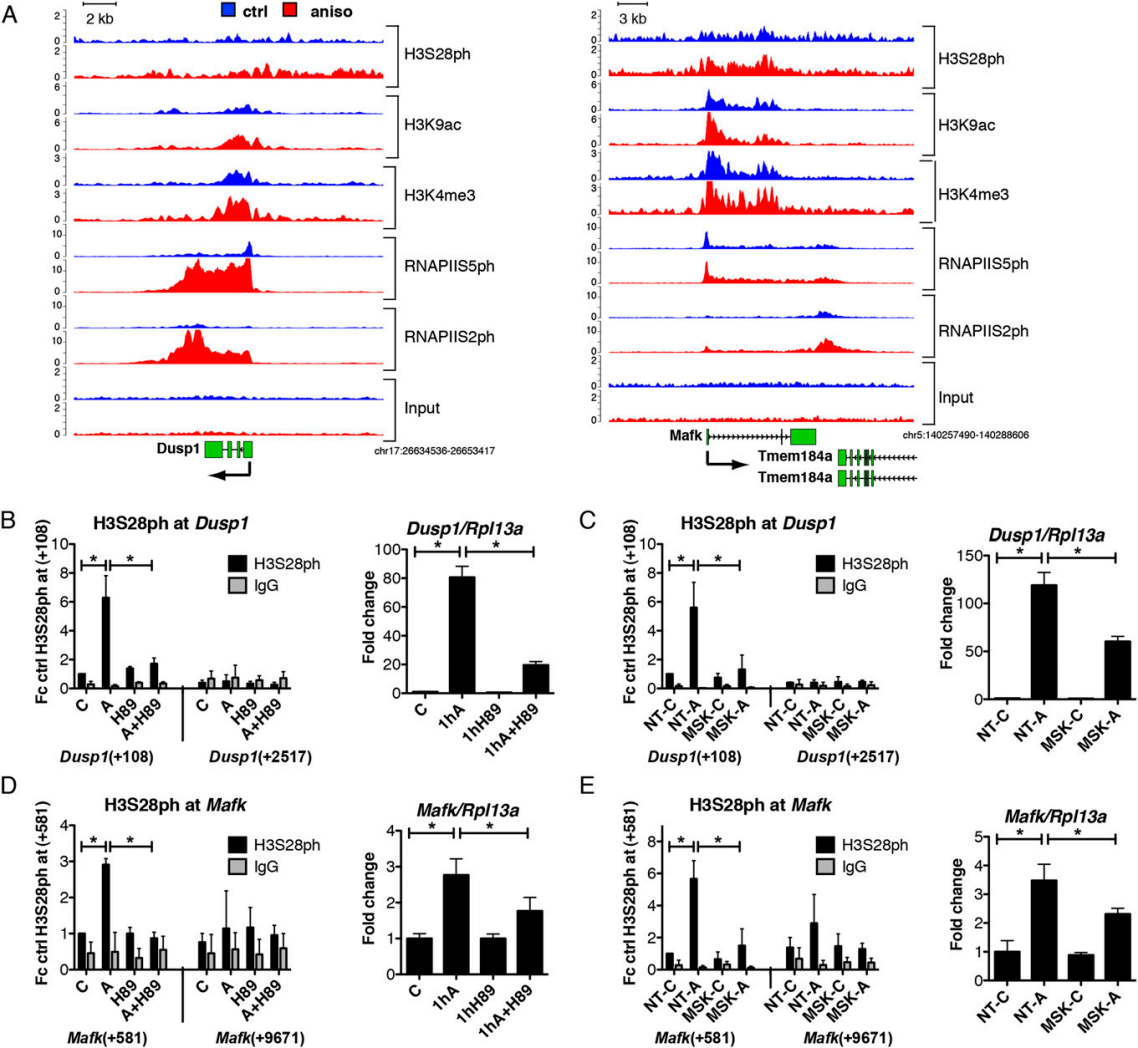
Stress-induced deposition of H3S28ph is dependent on MSK1/2 activity. (*A*) Genome browser representations of H3S28ph, H3K9ac, H3K4me3, RNAPIIS5ph, and RNAPIIS2ph normalized tag density profiles of representative genes (*Dusp1* and *Mafk)* up-regulated after 1 h of treatment with anisomycin. The profiles derived from untreated cells are depicted in blue and profiles of cells under stress-induced conditions are shown in red. (*B*,*D*) ChIP-qPCR analysis of stress-induced H3S28ph levels upon MSK1/2 inhibition with H89 at *Dusp1* and *Mafk* genes (*left* panels); and RT-qPCR analysis of mRNA expression of *Dusp1* and *Mafk* genes (*right* panels) in control (C) and anisomycin-treated cells (A) in the absence or presence of H89. Error bars represent SDs (*n* = 3). (*) *P* < 0.05. (*C*,*E*) ChIP-qPCR analysis of stress-induced H3S28ph in knockdown control (NT) and *Rpska5* knockdown (MSK) cells at *Dusp1* and *Mafk* genes (*left* panels); and RT-qPCR analysis of mRNA expression of *Dusp1* and *Mafk* genes (*right* panels) upon anisomycin treatment in knockdown control (NT) and *Rpska5* knockdown (MSK) cells. Error bars represent SDs (*n* = 3). (*) *P* < 0.05. Knockdown efficiency is shown in Supplemental Figure 10B–D.

Given the fact that histone H3S10 and H3S28 phosphorylation has been linked to the activation of IE genes in several biological systems (Gehani et al. 2010; Lau and Cheung 2011), we next asked what proportion of up-regulated genes coincides with the presence of H3S28ph mark after 1 h of anisomycin treatment. The analysis of H3S28ph ChIP-seq revealed 138 up-regulated genes (49% of all up-regulated genes, *P*-value = 3.9 × 10^−55^, hypergeometric test) associated with nucleosomes carrying H3S28ph. As shown for four representative genes (*Traf1*, *Dusp1*, *Mafk*, and *Nfil3)* transcriptional activation in response to stress correlated with increased H3S28ph signals in the promoter regions, accompanied by increased H3K9ac and H3K4me3 marks (Fig. 3A; Supplemental Fig. 9). The effector kinases MSK1 (encoded by *Rps6ka5*) and MSK2 (encoded by *Rps6ka4*) have been shown to phosphorylate H3S28 upon stress and growth factor stimulation (Soloaga et al. 2003). H89 is a potent inhibitor of MSK1/2 (Edmunds and Mahadevan 2004) that efficiently blocks stress-induced deposition of H3S28ph (Supplemental Fig. 10A). In order to determine whether stress-induced phosphorylation of H3S28 at activated genes depends on MSK1/2 activity, we performed ChIP-qPCR analysis of H3S28ph levels at selected loci in H89-treated cells. As expected, chemical inhibition of MSK1/2 activity by H89 greatly reduced phosphorylation of H3S28 at the promoters of target genes upon stress stimulation (Fig. 3B,D; Supplemental Fig. 10E,G, left panels). Consistent with this, cells transfected with siRNAs against *Rps6ka4* and *Rpska5* showed the same effect (Figs. 3C,E; Supplemental Fig. 10F,H, left panels), further demonstrating that stress-induced accumulation of H3S28ph depends on MAP kinase signaling via MSKs. Since *Rps6ka4* (MSK1) knockdown affected the protein levels only slightly, we refer to those cells as MSK2 knockdown cells. Importantly, the reduction in MSK2 protein was sufficient to greatly abolish global levels of H3S28ph upon stress stimulation (Supplemental Fig. 10B–D). This is in agreement with earlier studies demonstrating that MSK2 is the major kinase responsible for anisomycin-induced H3S28 phosphorylation in fibroblasts (Soloaga et al. 2003). Moreover, both chemical inhibition of MSK activity with H89, as well as siRNA-mediated knockdown of *Rpska5*, reduced the induction of these genes upon anisomycin treatment (Figs. 3B–E; Supplemental Fig. 10E–H, right panels). In addition, H89 treatment interferes with the increased occupancy of RNAPIIS5ph and RNAPIIS2ph upon stress stimulation (Supplemental Fig. 11), suggesting a contribution of MSK1/2 mediated H3S28 phosphorylation to transcriptional activation.

### Transcription of H3S28-targeted genes is regulated at the level of RNAPII initiation and elongation

**Figure 4.**
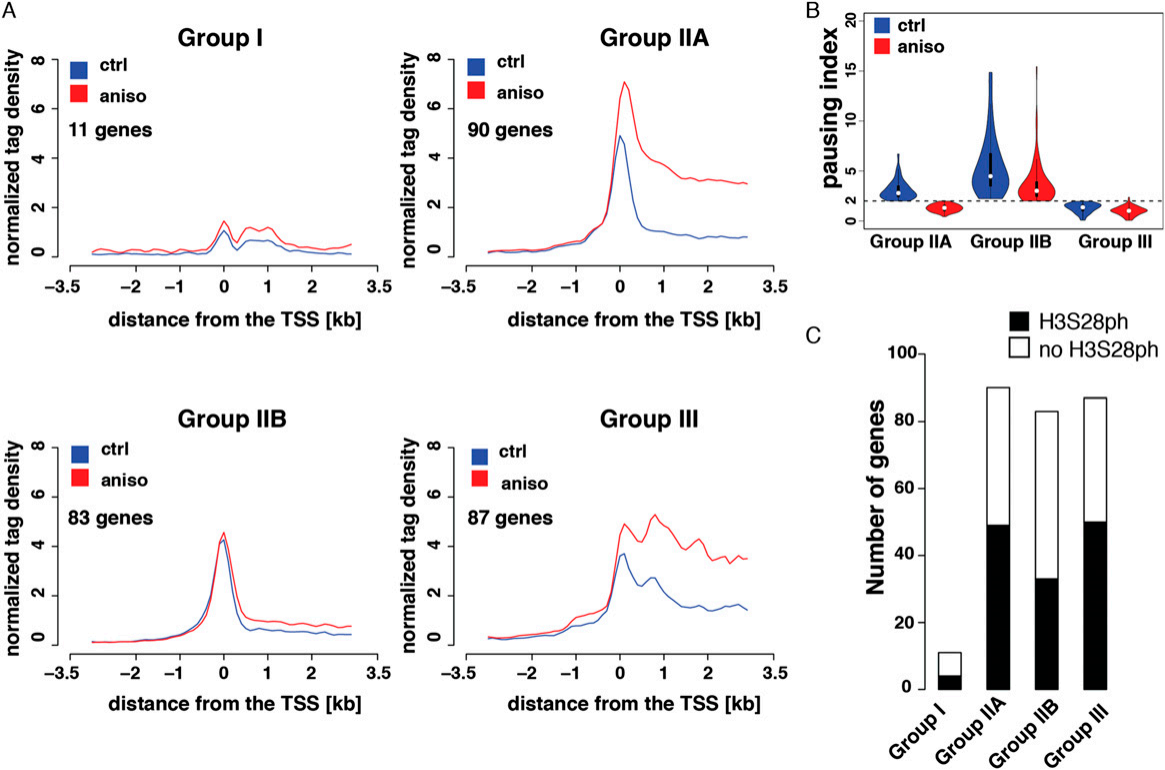
Classification of stress-induced genes. (*A*) Normalized RNAPIIS5ph ChIP-seq tag density profiles of untreated (blue line) and stress-induced (red line) serum-deprived mouse Swiss 3T3 fibroblasts. The genes are divided into four groups according to their pausing indices and the presence of scRNA. (*B*) Violin plots showing the distribution of pausing indices among groups of genes associated with different RNAPII profiles. (*C*) Histogram showing the distribution of H3S28ph-marked genes among different regulatory groups. None of the groups is significantly enriched in H3S28ph marked genes (*P*-value = 0.07, Fisher’s exact test).

An increasing body of evidence demonstrates that expression of stimulus-responsive genes in metazoans is often regulated at the transition of the RNAPII complex from the promoter–proximal paused to the elongation-competent state (Rasmussen and Lis 1993; Muse et al. 2007; Core et al. 2008; Rahl et al. 2010). Moreover, the vast majority of genes harboring promoter–proximal paused RNAPII is transcriptionally active in basal conditions (Guenther et al. 2007; Zeitlinger et al. 2007; Core et al. 2008; Gilchrist et al. 2010; Rahl et al. 2010; Min et al. 2011). Importantly, the phosphorylation of H3S10 upon mitogen stimulation has previously been linked to the release of promoter–proximal paused polymerase complexes (Zippo et al. 2009). In order to determine whether expression of stress-induced target genes is regulated at the post-initiation step of their transcription, we complemented the genome-wide studies of initiation-engaged (RNAPIIS5ph) and elongation-competent (RNAPIIS2ph) RNAPII with the analysis of short-capped RNAs (scRNAs). scRNAs are nascent transcripts associated with the transcriptionally engaged but paused RNAPII complexes and are characterized by the presence of a 7-methylguanosine cap on their 5′ end (Rasmussen and Lis 1993). To address whether paused RNAPII complexes are present at promoters of stress-induced genes prior to their activation, we isolated and sequenced these RNA species using a strategy previously developed for *Drosophila* (Nechaev et al. 2010) (Supplemental Fig. 12A–C). As previously described (Nechaev et al. 2010), scRNA abundance correlated well with the amount of RNAPIIS5ph around the TSSs (Supplemental Fig. 12D). In order to identify promoter–proximal paused RNAPII in our stress-induced system, we calculated pausing indices for each gene as the ratio of the RNAPIIS5ph signal around the TSS to the signal in gene body. This parameter was previously demonstrated to be a good estimate of the efficiency of RNAPII release into productive elongation (Muse et al. 2007; Zeitlinger et al. 2007). In agreement with this, pausing index and scRNA abundance correlated well (Supplemental Fig. 12E). Genes showing a pausing index higher than two and characterized by the presence of scRNA were subsequently regarded as paused. Based on the RNAPII status in control and anisomycin-treated cells we classified the stress-induced genes into four groups (Fig. 4A). Group I comprises up-regulated genes that showed no detectable expression in the control state (log_2_ RPKM mRNA-seq < 0), no association with promoter–proximal paused polymerase, and no scRNAs expression. Genes in Group IIA are characterized by promoter–proximal paused polymerase in the control state. These genes show a decrease in the pausing index upon stress stimulation, indicating efficient release of RNAPII into productive elongation (pausing index < 2 upon anisomycin treatment) (Fig. 4B). Group IIB also comprises genes classified as paused in the control state, which show an increase in mRNA expression upon stress induction. However, despite the decrease in their pausing index upon anisomycin treatment (Fig. 4B), they remain associated with promoter–proximal paused polymerase (pausing index > 2 upon anisomycin treatment). Group III contains up-regulated genes that are expressed in untreated cells and are not associated with promoter–proximal paused polymerase. The four groups are represented by the genes *Traf1* (Group I), *Dusp1* (Group IIA), *Mafk* (Group IIB), and *Nfil3* (Group III) shown in Figure 3A and Supplemental Figure 9. Taken together, our analysis revealed that most of the stress-induced genes are regulated at the post-initiation step of their transcriptional cycle. However, importantly, the prevalence of stress-induced H3S28ph was not particularly associated with any of the four groups (Fig. 4C, *P*-value = 0.07, Fisher’s exact test).

### H3S28 phosphorylation primes a subset of genes for future induction

**Figure 5.**
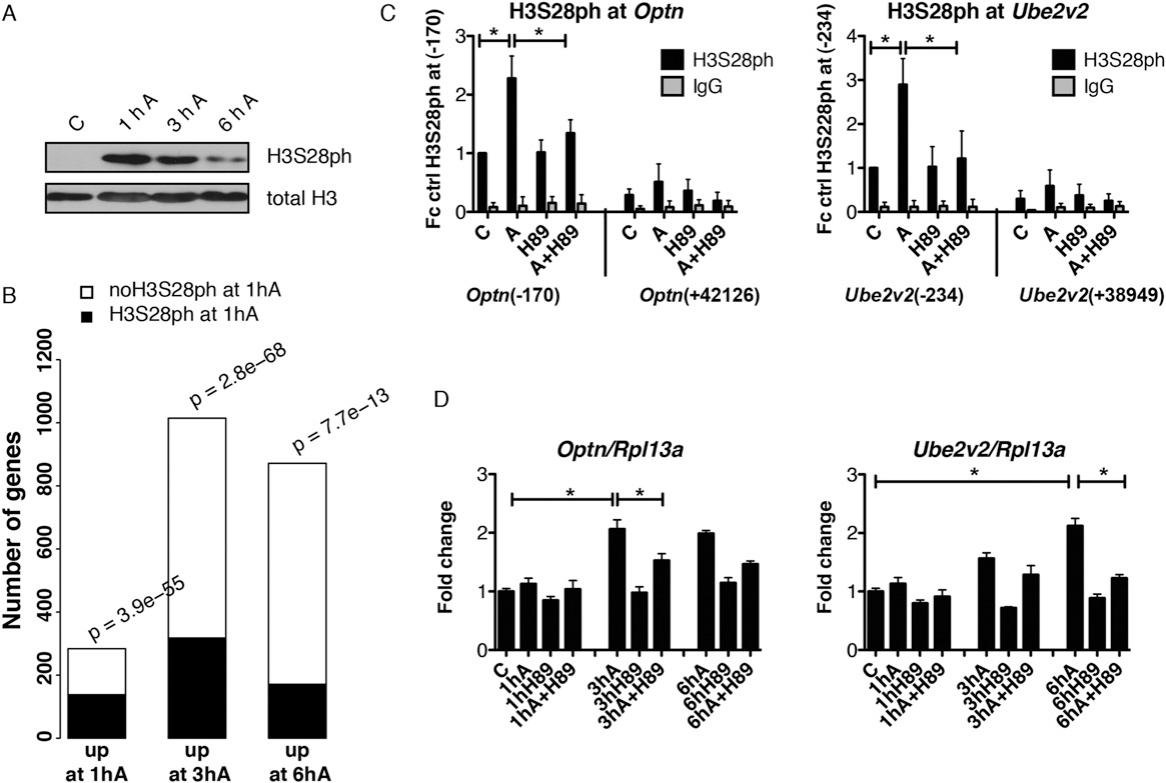
A subset of H3S28ph target genes is primed for later activation. (*A*) Western blot analysis of H3S28ph levels in serum deprived mouse Swiss 3T3 fibroblasts treated with anisomycin for 1, 3, and 6 h. H3 C-terminus antibody was used as a loading control. (*B*) Barplot showing the overlap between genes associated with H3S28ph after 1 h of anisomycin treatment and genes up-regulated after 1, 3, and 6 h after stress induction. *P*-values were calculated using a hypergeometric test. (*C*) ChIP-qPCR analysis of stress-induced H3S28ph at *Optn* and *Ube2v2* genes in control (C) and anisomycin-treated cells (A) in the absence or presence of H89. Error bars represent SDs (*n* = 3). (*) *P* < 0.05. (*D*) RT-qPCR analysis of *Optn* and *Ube2v2* gene expression after 1, 3, and 6 h of anisomycin treatment in the absence and presence of H89. Error bars represent SDs (*n* = 3). (*) *P* < 0.05.

Transcriptional response to external stimuli comprises target genes with distinct induction kinetics, where products of the early phase of the response often encode factors that regulate activation of the genes induced at the later stages (Hargreaves et al. 2009). Importantly, stress-induced H3S28ph is only transiently deposited, as its total abundance and gene-associated levels decreased after 3 and 6 h of anisomycin treatment (Fig. 5A; Supplemental Fig. 13). We therefore asked whether early deposition of H3S28ph contributes to the expression of genes that require longer stimulation for their expression. We therefore performed a microarray analysis of serum-deprived Swiss 3T3 fibroblasts treated with anisomycin for 3 and 6 h. We identified 1015 genes with an increased expression level only after 3 h of stimulation (fold change > 2 after a 3-h treatment but not after 1 h of anisomycin treatment and *P* < 0.05, referred to as 3hA targets) and 872 genes up-regulated only after 6 h of treatment (fold change > 2 after 6 h of treatment but not after 3 h or 1 h of anisomycin treatment and *P* < 0.05, referred to as 6hA targets). Notably, 31% of 3hA targets and 20% of 6hA targets already carry an H3S28ph mark after 1 h of anisomycin treatment (Fig. 5B, *P* = 2.8 × 10^−68^ and *P* = 7.7 × 10^−13^, respectively, hypergeometric test). In order to determine whether MSK1/2 activity is required for activation of these genes, we chemically inhibited the kinases with H89 and analyzed expression levels of the 3hA targets *Optn, Ell*, and *Tank* and the 6hA target *Ube2v2*, which show the presence of H3S28ph after 1 h of anisomycin treatment (Fig. 5C; Supplemental Fig. 14A). Indeed, inhibition of MSK1/2 decreased the induction of these genes (Fig. 5D; Supplemental Fig. 14B). In order to determine whether the activation of genes at later time points requires MSK1/2 activity, we performed an mRNA-seq analysis of cells treated with anisomycin for 3 or 6 h in the presence or absence of H89 inhibitor. To exclude the possible effects of the inhibitor alone on the gene expression profiles, mRNA of cells treated for 3 and 6 h only with H89 was analyzed. Biological replicates of all the experiments correlated well (Supplemental Fig. 15). Strikingly, 93% of 3hA targets and 87% of 6hA targets showed reduced induction upon MSK1/2 inhibition (Supplemental Table 6). Taken together, this indicates that even though for many H3S28ph target genes the presence of this mark does not coincide with a simultaneous change in their expression level, it may contribute to the activation of a subset of genes that exhibit slower induction kinetics.

### Stress-induced H3S28ph correlates with the increase in local histone acetylation levels

**Figure 6.**
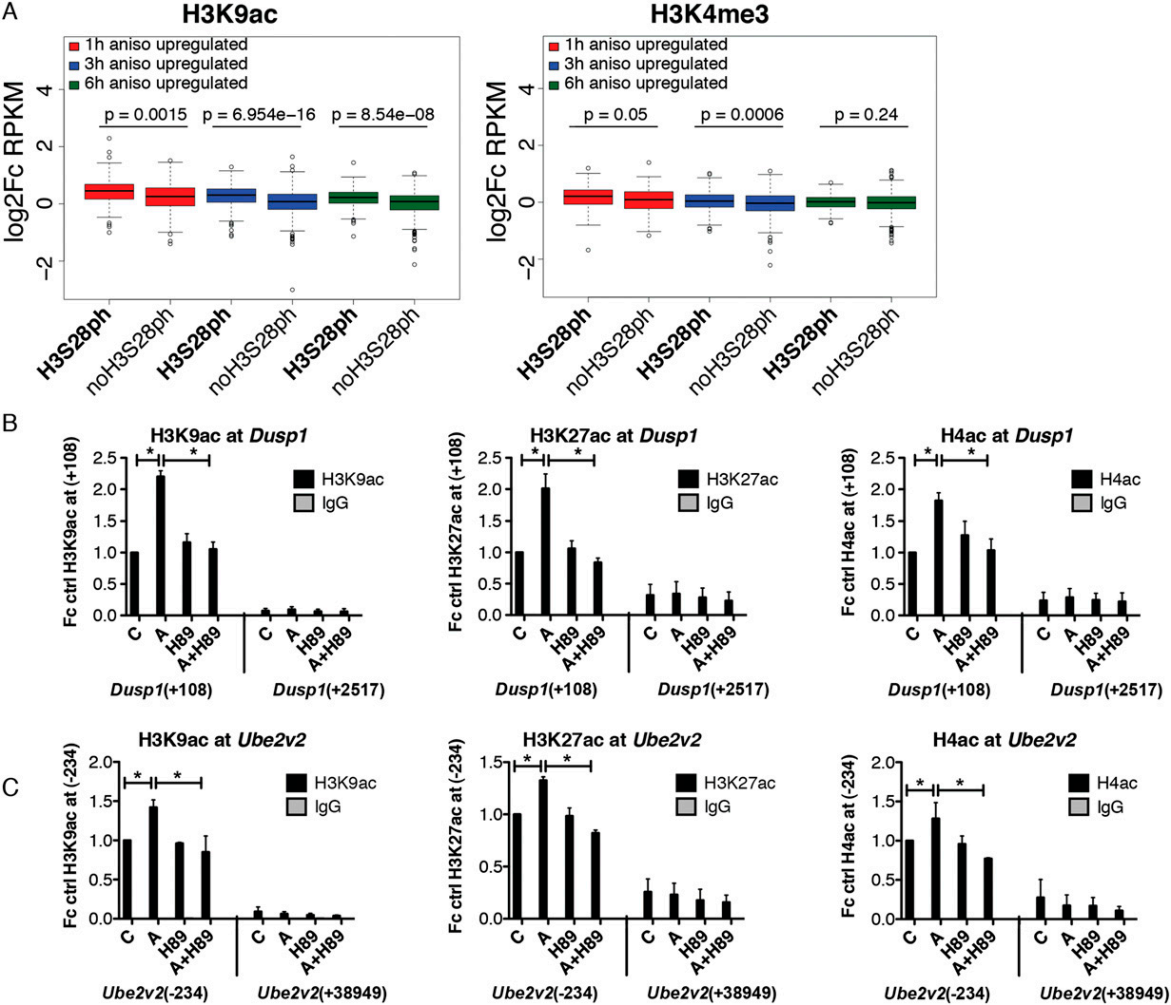
H3S28ph-marked genes show higher increase in histone acetylation levels upon 1 h of anisomycin stimulation. (*A*) Boxplots showing log_2_ fold change in normalized read counts (RPKM) for H3K9ac and H3K4me3 ChIP-seq after 1 h of treatment with anisomycin at 1hA, 3hA, and 6hA targets. Fold change was calculated as the ratio of RPKM in anisomycin condition to RPKM in the control state at the regions from −1 kb to +3 kb surrounding the TSS. *P*-values were determined by the Mann-Whitney *U* test. (*B,C*) ChIP-qPCR analysis of H3K9ac, H3K27ac, and H4ac levels at *Dusp1* and *Ube2v2* genes in control (C) and anisomycin-treated cells (A) in the absence and presence of H89. Error bars represent SDs (*n* = 3). (*) *P* < 0.05.

Given the link between histone phosphorylation and histone acetylation (Cheung et al. 2000b; Thomson et al. 2001), we analyzed H3K9ac and H3K4me3 at H3S28ph-marked genes after 1 h of treatment with anisomycin. Interestingly, for genes induced at each time point studied, we observed a higher increase in acetylation at genes carrying H3S28ph than genes that lack this modification after 1 h of treatment (Fig. 6A, left panel). In contrast, H3K4me3 did not follow this pattern, as there was no difference in H3K4me3 between 6hA targets that show the presence of H3S28ph after 1 h of anisomycin stimulation and the ones that did not (Fig. 6A, right panel). Importantly, the increase in histone acetylation after 1 h of anisomycin treatment was sensitive to inhibition of MSK1/2. Treatment with H89 abolished the increase in H3K9ac, H3K27ac, and H4ac at 1hA targets (*Dusp1*, *Mafk*) as well as at 3hA and 6hA target genes (*Optn*, *Ell*, *Tank*, *Ube2v2*) (Fig. 6B,C; Supplemental Fig. 16). An increase in H3K4me3 after 1 h of anisomycin treatment was only observed for 1hA targets and was prevented by H89 treatment (Supplemental Fig. 17).

### H3S28ph modulates the recruitment of HDAC-containing complexes

**Figure 7.**
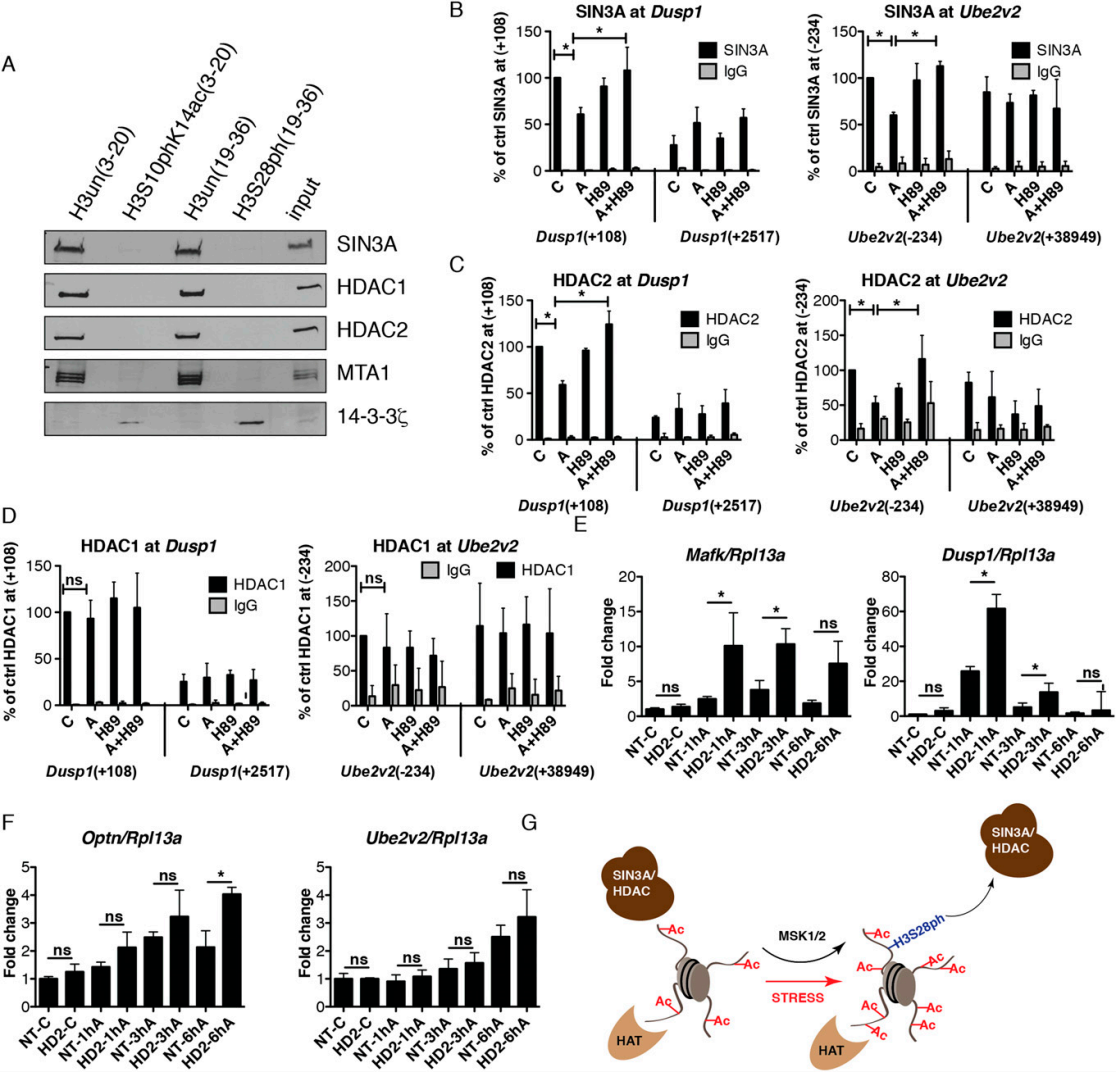
H3S28ph mediates the dissociation of HDAC-containing complexes from target promoters. (*A*) Western blot analysis of histone pull-down assays with nuclear extracts from HeLa cells treated with anisomycin for 1 h and synthetic peptides corresponding to aa 3–20 and 19–36 of histone H3, either unmodified or carrying the phosphorylation mark at S10 or S28. The association of SIN3A, HDAC1, HDAC2, MTA1, and 14-3-3 zeta (encoded by *Ywhaz*) with differentially modified peptides was analyzed. (*B–D*) ChIP-qPCR analysis of changes in SIN3A, HDAC2, and HDAC1 occupancy at *Dusp1* and *Ube2v2* genes in control (C) and anisomycin-treated cells (A) in the absence and presence of H89. Error bars represent SDs (*n* = 3). (*) *P* < 0.05. (*E*,*F*) RT-qPCR analysis of *Dusp1*, *Mafk*, *Ube2v2*, and *Optn* gene expression upon anisomycin treatment (A) in control (NT) and *Hdac2* (HD2) knockdown cells. Error bars represent SDs (*n* = 3). (*) *P* < 0.05. (*G*) A model demonstrating the impact of H3S28 phosphorylation on the local histone acetylation levels at stress-induced genes. Local histone acetylation results from the dynamic interplay between recruited HAT and HDAC activities. Upon stress stimulation, MSK1/2 phosphorylates S28 at histone H3 at stress target promoters. This leads to dissociation of HDAC-containing complexes, thereby inducing an increase in local histone acetylation levels.

In order to identify factors whose binding to histone H3 tail is sensitive to the phosphorylation status of S28 residue, we carried out peptide pull-down assays using synthetic peptides (19–36 aa peptides, either phosphorylated at S28 or unmodified) and nuclear extracts from HeLa cells treated with anisomycin for 1 h. Bound factors were subsequently analyzed by mass spectrometry. As previously described (Winter et al. 2008a), we found a specific binding of 14-3-3 proteins to the H3S28 phosphorylated peptides (Supplemental Fig. 18; the complete set of identified proteins is reported in Supplemental Tables 7–9). Interestingly, we found several components of the Sin3A and NuRD corepressor complexes associated with the unmodified but not with the H3S28ph histone H3 peptides (Supplemental Fig. 18). The interaction with the Sin3A complex components SIN3A, HDAC1, and HDAC2, and the NuRD complex component MTA1, was confirmed by Western blot analysis of independent pull-down experiments (Fig. 7A). Peptides corresponding to the amino acid sequence 3–20 of histone H3 served as a control, since phosphorylation of S10 has been shown to influence HDAC1 and HDAC2 binding (He et al. 2013).

In order to determine whether H3S28ph influences the interaction of HDAC-containing complexes with chromatin in vivo, we performed ChIP-qPCR analysis of SIN3A, HDAC1, and HDAC2 binding to the stress-induced target genes. We observed a reduction in SIN3A and HDAC2 binding in response to stress (Fig. 7B,C; Supplemental Fig. 19). This effect was abolished in the presence of the MSK1/2 inhibitor H89, suggesting that stress-induced H3S28 phosphorylation regulates the association of HDAC-containing corepressor complexes with chromatin, thereby modulating local histone acetylation levels. Although HDAC1 bound the unmodified peptide but not the phosphorylated one (Fig. 7A), we did not detect any consistent reduction in HDAC1 occupancy at stress-induced target genes upon anisomycin treatment (Fig. 7D; Supplemental Fig. 19). Histone acetylation levels are regulated by the opposing activities of histone acetyltransferases (HAT) and HDACs (Kurdistani and Grunstein 2003). Several HATs have been reported to play a role in signal-inducible transcription. We therefore analyzed the recruitment of EP300, KAT2A, and KAT2B at the target genes; however, we did not observe a consistent increase in association of these chromatin modifiers (Supplemental Fig. 20). In order to determine the consequences of the reduction in HDAC occupancy for expression of the stress-induced genes, we performed shRNA-mediated knockdown of *Hdac2* (Supplemental Fig. 21A). Strikingly, *Hdac2* knockdown cells showed much higher induction of *Dusp1* and *Mafk* expression upon anisomycin treatment (Fig. 7E). The reduction in HDAC2 did not affect the expression of these genes under basal conditions. This is in agreement with previous studies in yeast demonstrating that the deletion of many chromatin modifiers does not generally influence steady-state transcription but affects transcriptional programs upon external signaling (Weiner et al. 2012). Interestingly, *Hdac2* knockdown had no effect on the expression of *Fos* and *Egr3—*two IE genes lacking the H3S28ph mark upon anisomycin treatment (Supplemental Fig. 21B). This demonstrates that reduction in HDAC2 does not alter the activation of all IE genes upon stress stimulation. *Hdac2* knockdown also resulted in enhanced activation of 3hA target genes *Optn* and *Ell*, albeit at later time points, whereas no effect was observed for *Ube2v2* and *Tank* expression (Fig. 7F; Supplemental Fig. 21C). This suggests that HDAC2 acts as a modulator of stress-induced transcription and the reduction in its levels, at least for a subset of genes, directly influences the transcriptional response to stress. In conclusion, we propose a model (Fig. 7G) in which stress-induced histone H3S28 phosphorylation reduces the binding of HDAC-containing complexes to chromatin, leading to a local increase in histone acetylation levels and subsequent transcriptional induction.

## Discussion

Here we report a genome-wide analysis of histone H3S28 phosphorylation in the context of stress signaling in mammalian cells. Importantly, we found that this modification targets as many as 50% of all IE stress-induced genes, indicating an important role of this histone mark in stress-induced transcription. Our findings provide new insights into the role of H3S28ph in transcriptional regulation and suggest a model where stress-induced histone H3S28 phosphorylation regulates the association of corepressor complexes with chromatin.

There are several possible mechanisms of how the dissociation of HDAC-containing complexes upon H3S28 phosphorylation influences the transcriptional status of a given genomic region. Here we show that the H3S28ph-mediated decrease in HDAC occupancy is accompanied by increased local histone acetylation. Enhanced histone acetylation was shown to destabilize nucleosome structure (Boeger et al. 2003), thus facilitating RNAPII binding (Schones et al. 2008). However, the increase in histone acetylation alone is not always sufficient for transcriptional induction, as histone deacetylase inhibitors activate only a subset of genes despite their global effect on histone acetylation levels (Glaser et al. 2003). Signaling cascades converge on transcription factors that define specificity of the cellular response (Johnson and Dent 2013). Since the repertoire and activity of transcription factors is cell type-specific and subject to regulation by external signals, the selection of genomic targets and their induction kinetics in a given pathway is largely dependent on the biological context. This at least partly explains why only a subset of H3S28ph target genes in serum-starved mouse Swiss 3T3 fibroblasts undergoes transcriptional activation. In agreement with this, H3S10 phosphorylation upon LPS treatment in dendritic cells was shown to mark promoters for the recruitment of NFkappaB (Saccani et al. 2002), which become activated upon inflammatory signaling, underlining cell type-specific interpretation of the signaling processes.

In addition, reversible acetylation was shown to modulate the activity of many transcription factors and components of the basal transcriptional machinery (Imhof et al. 1997; Soutoglou et al. 2000; Schroder et al. 2013). Since SIN3A-mediated deacetylation of MYC has been demonstrated to trigger the degradation of this transcription factor (Nascimento et al. 2011), it is likely that the dissociation of HDAC-containing complexes upon H3S28 phosphorylation constitutes a convenient mechanism to modulate transcription by inducing local changes in acetylation of chromatin-bound factors, thereby regulating their activity. Given that MSK1/2 were shown to phosphorylate other factors besides histone H3 (Wiggin et al. 2002), we cannot exclude the possibility that such modifications, in addition to H3S28ph, play a role in transcriptional induction of stress-induced genes.

MAPK-induced phosphorylation of histone H3 has been shown to exert a negative effect on effector binding. Upon serum or anisomycin stimulation, MSK1-mediated phosphorylation leads to dissociation of the repressor HP1g (encoded by *Cbx3*) from *HDAC1* gene promoter, resulting in transcriptional activation (Winter et al. 2008b). Similarly, phosphorylation of S28 shows a negative impact on the Polycomb repressive complex association with mitotic chromosomes (Fonseca et al. 2012) as well as with several gene promoters upon MAPK activation in interphase (Gehani et al. 2010; Lau and Cheung 2011). The data presented here establish histone H3S28 phosphorylation as an important signal-induced chromatin modification that modulates the association of corepressor complexes with their target promoters.

## Methods

### Cell culture

Mouse Swiss 3T3 fibroblasts and HeLa cells were cultured as described previously (Winter et al. 2008b). Swiss 3T3 fibroblasts were arrested in G_0_ phase of the cell cycle by serum deprivation for 72 h using DMEM containing 0.2% FCS (vol/vol). Resting cells were treated with 188.5 nM anisomycin (Sigma-Aldrich) for 1 h. The following inhibitors were used in this study: 10 μM H89 (Santa Cruz Biotechnology, 15-min pretreatment), 10 μM SB203580 (Santa Cruz Biotechnology, 30-min pretreatment), triptolide 1 μM (Tocaris, 1-h pretreatment), 0.2 μg/mL actinomycin D (Sigma, 1-h pretreatment).

### Total cellular RNA isolation and real-time PCR (RT-qPCR)

Total RNA was isolated using TRIzol reagent (Invitrogen) according to the manufacturer’s instructions. One microgram of RNA was reversely transcribed with the *iScript* cDNA Synthesis Kit (Bio-Rad) and 1:20 dilution of cDNA was analyzed in real-time PCR with the KAPA SYBR FAST qPCR kit (Peqlab) on the iCycler IQ system (Bio-Rad). Primer sequences are listed in Supplemental Material. Housekeeping gene ribosomal protein L13a (*Rpl13a*) was used for the normalization. Statistical significance of the observed changes was determined using one-way ANOVA followed by the Tukey HSD post-hoc test, both implemented in R (R Development Core Team 2014).

### siRNA-mediated and shRNA-mediated knockdown

For siRNA-mediated knockdown, 3 × 10^5^ (or 3 × 10^6^) Swiss 3T3 fibroblasts were seeded in a 3.5-cm (or 15-cm) dish in DMEM supplemented with 10% FCS without antibiotics, and on the following day the medium was replaced with DMEM containing 0.2% FCS and no antibiotics. The next day the medium was replaced again (with DMEM containing 0.2% FCS and no antibiotics) and cells were transfected with 50 pmol (300 pmol) ON-TARGETplus SMART pool siRNA (Dharmacon) using Lipofectamine RNAiMAX Reagent (Invitrogen) in OptiMEM (Invitrogen). After 48 h the cells were treated with anisomycin. shRNA-mediated knockdown of *Hdac2* was performed as described previously (Lagger et al. 2010).

### Western blot analysis

Histone isolation, whole-cell extract isolation, and Western blotting were performed as described (Hauser et al. 2002; Lagger et al. 2010). Dot blots were performed as described previously (Brunmeir et al. 2010). The antibodies used to detect proteins/peptides are listed below.

### mRNA sequencing

Ten micrograms of total RNA was subjected to two rounds of poly(A) selection with the Dynabeads mRNA Purification kit (Invitrogen). mRNA was subsequently fragmented by hydrolysis (40 mM TrisOAc at pH 8.2, 100 mM KOAc, 150 mM MgOAc) at 94°C for 3 min. First-strand cDNA synthesis was performed using the SuperScript III Reverse Transcriptase kit (Invitrogen) with random hexamers priming (Applied Biosystems) in the presence of actinomycin D (5 ng/μL). Second-strand cDNA was synthesized using DNA Pol I, DNA ligase (both Invitrogen), and RNase H (NEB) with random hexamers (Applied Biosystems) in the presence of dUTP. The libraries were prepared by the Vienna Biocenter CSF NGS unit using the NEBNext Library Prep Reagent Set for Illumina (NEB), multiplexed (2 samples/lane), and sequenced on HiSeq 2000 (Illumina) at the CSF NGS unit (50 bp single-end reads). Reads were mapped to the mouse genome (NCBI37/mm9 annotation from July 2007) using TopHat (Trapnell et al. 2009) v1.4.1 allowing for one mismatch per 18-bp segment and retaining only uniquely mapped reads. Differential expression was performed using htseq-count script (Anders 2010) with the “union” model and the Bioconductor package edgeR (Gentleman et al. 2004; Robinson et al. 2010). A detailed description of differential expression analysis is provided in Supplemental Material. The GO enrichment analysis was performed using DAVID (Huang da et al. 2009). Known transcription-factor motif enrichment analysis in promoters (−400 bp to +100 bp from the TSS) was performed using HOMER (Heinz et al. 2010) findMotifsGenome.pl script. Promoter sequences of noninduced genes served as a background for differential enrichment analysis. *P*-values were determined by a hypergeometric test.

### Chromatin immunoprecipitation and chromatin immunoprecipitation followed by sequencing (ChIP-seq)

Chromatin immunoprecipitation was performed as previously described (Hauser et al. 2002) with the following modifications: Chromatin-antibody complexes were pulled down using Dynabeads protein A or G beads (Invitrogen) and the amount of extracted DNA was analyzed using KAPA SYBR FAST qPCR kit (Peqlab) on the iCycler IQ system (Bio-Rad), and the amount of immunoprecipitated DNA was calculated as the % of input (1:20 dilution of genomic DNA). ChIP signals for histone modifications were normalized to the H3 C terminus signal to correct for changes in nucleosomal density. For all experiments, the ChIP signal in the control condition in one of the tested gene regions was set to 100% (in the case of HAT and HDAC occupancy) or to 1 (in the case of chromatin modifications and RNAPII). Statistical significance of observed changes was determined using a one-sample *t*-test (in the case of comparisons with the control) or a two-sample *t*-test (in the case of comparisons between different treatments) implemented in R. The *P*-values were corrected for multiple testing using the Hochberg method implemented in R. Primer sequences are listed in Supplemental Material. The libraries were prepared by the CSF NGS unit using the NEBNext Library Prep Reagent Set for Illumina (NEB) and sequenced either on GAIIx or HiSeq 2000 (Illumina) at the CSF NGS unit (36- or 50-bp single-end reads, Supplemental Table 1). Reads were mapped to the mouse genome (NCBI37/mm9 annotation from July 2007) using Bowtie version 12.5 (Langmead et al. 2009), allowing up to two mismatches and retaining reads that map to only one genomic location. Peak calling was performed using MACS v1.4.4 (Zhang et al. 2008) with a *P*-value cutoff of 1 × 10^10^ and a shiftsize of 100. Peaks were assigned to genes based on RefSeq gene annotation (Pruitt et al. 2007) using the ChIPpeakAnno Bioconductor package (Zhu et al. 2010). Sequencing tracks were visualized with the Gviz R package (Hahne et al. 2013). A detailed description of ChIP-seq analysis is provided in Supplemental Material. In the case of HDAC1, HDAC2, and SIN3A ChIPs, the cells were first crosslinked with 2 mM disuccinimidyl glutarate (Applichem) for 30 min at room temperature prior to crosslinking with formaldehyde.

### Nuclear RNA isolation and short-capped RNA (scRNA) sequencing

In brief, the cells were first resuspended in Solution I (150 mM KCl, 4 mM MgOAc, 10 mM Tris-HCl at pH 7.4) and spun down for 5 min at 500*g*, 4°C. The pellet was resuspended in Solution II (Solution I + 0.5% NP40) and incubated on ice for 10 min. Nuclei were pelleted through a cushion of 0.6 M sucrose at 900*g* for 10 min, 4°C. Following the removal of the supernatant, TRIzol reagent (Invitrogen) was added and RNA was further isolated according to the total RNA isolation protocol. scRNA-seq was performed as described (Nechaev et al. 2010), with modifications allowing for the paired-end sequencing. The adapter sequences as well as primer sequences for library generation are listed in the Supplemental Material. The libraries were sequenced on either GAII (76 bp reads paired-end, first experiment) or HiSeq 2000 (100 bp reads paired-end, second experiment) (Illumina) at the CSF NGS unit. Reads were first trimmed to 25 bp and then mapped to the mouse genome using the strategy previously described for mRNA-seq. Detailed description of scRNA-seq analysis is provided in Supplemental Material.

### Nuclear extract isolation for histone peptide pull-down assay and mass spectrometry

Nuclear extract isolation and histone peptide pull-down assay was performed as described previously (Winter et al. 2008b). Peptide sequences are provided in Supplemental Material. Mass spectrometry analysis was performed as described previously (Kocher et al. 2012). A detailed protocol is provided in the Supplemental Material.

## Data access

The sequencing and microarray data have been submitted to the NCBI Gene Expression Omnibus (GEO; http://www.ncbi.nlm.nih.gov/geo/) under accession number GSE55784.

## References

[B1] Anders S. 2010. *HTSeq: Analysing high-throughput sequencing data with Python*. EMBL Heidelberg (Genome Biology Unit), Heidelberg, Germany

[B2] Banerjee T, Chakravarti D. 2011. A peek into the complex realm of histone phosphorylation. Mol Cell Biol 31: 4858–4873 22006017 10.1128/MCB.05631-11 PMC3233023

[B3] Basehoar AD, Zanton SJ, Pugh BF. 2004. Identification and distinct regulation of yeast TATA box-containing genes. Cell 116: 699–709 15006352 10.1016/s0092-8674(04)00205-3

[B4] Boeger H, Griesenbeck J, Strattan JS, Kornberg RD. 2003. Nucleosomes unfold completely at a transcriptionally active promoter. Mol Cell 11: 1587–1598 12820971 10.1016/s1097-2765(03)00231-4

[B5] Brunmeir R, Lagger S, Simboeck E, Sawicka A, Egger G, Hagelkruys A, Zhang Y, Matthias P, Miller WJ, Seiser C. 2010. Epigenetic regulation of a murine retrotransposon by a dual histone modification mark. PLoS Genet 6: e1000927 20442873 10.1371/journal.pgen.1000927 PMC2861705

[B6] Cheung P, Allis CD, Sassone-Corsi P. 2000a. Signaling to chromatin through histone modifications. Cell 103: 263–271 11057899 10.1016/s0092-8674(00)00118-5

[B7] Cheung P, Tanner KG, Cheung WL, Sassone-Corsi P, Denu JM, Allis CD. 2000b. Synergistic coupling of histone H3 phosphorylation and acetylation in response to epidermal growth factor stimulation. Mol Cell 5: 905–915 10911985 10.1016/s1097-2765(00)80256-7

[B8] Clayton AL, Mahadevan LC. 2003. MAP kinase-mediated phosphoacetylation of histone H3 and inducible gene regulation. FEBS Lett 546: 51–58 12829236 10.1016/s0014-5793(03)00451-4

[B9] Core LJ, Waterfall JJ, Lis JT. 2008. Nascent RNA sequencing reveals widespread pausing and divergent initiation at human promoters. Science 322: 1845–1848 19056941 10.1126/science.1162228 PMC2833333

[B10] Deacon K, Mistry P, Chernoff J, Blank JL, Patel R. 2003. p38 Mitogen-activated protein kinase mediates cell death and p21-activated kinase mediates cell survival during chemotherapeutic drug-induced mitotic arrest. Mol Biol Cell 14: 2071–2087 12802076 10.1091/mbc.E02-10-0653 PMC165098

[B11] Drobic B, Perez-Cadahia B, Yu J, Kung SK, Davie JR. 2010. Promoter chromatin remodeling of immediate-early genes is mediated through H3 phosphorylation at either serine 28 or 10 by the MSK1 multi-protein complex. Nucleic Acids Res 38: 3196–3208 20129940 10.1093/nar/gkq030 PMC2879512

[B12] Edmunds JW, Mahadevan LC. 2004. MAP kinases as structural adaptors and enzymatic activators in transcription complexes. J Cell Sci 117: 3715–3723 15286173 10.1242/jcs.01346

[B13] Escoubet-Lozach L, Benner C, Kaikkonen MU, Lozach J, Heinz S, Spann NJ, Crotti A, Stender J, Ghisletti S, Reichart D, . 2011. Mechanisms establishing TLR4-responsive activation states of inflammatory response genes. PLoS Genet 7: e1002401 22174696 10.1371/journal.pgen.1002401 PMC3234212

[B14] Fonseca JP, Steffen PA, Muller S, Lu J, Sawicka A, Seiser C, Ringrose L. 2012. In vivo Polycomb kinetics and mitotic chromatin binding distinguish stem cells from differentiated cells. Genes Dev 26: 857–871 22508729 10.1101/gad.184648.111 PMC3337459

[B15] Gao J, Wagnon JL, Protacio RM, Glazko GV, Beggs M, Raj V, Davidson MK, Wahls WP. 2013. A stress-activated, p38 mitogen-activated protein kinase-ATF/CREB pathway regulates posttranscriptional, sequence-dependent decay of target RNAs. Mol Cell Biol 33: 3026–3035 23732911 10.1128/MCB.00349-13 PMC3719685

[B16] Gehani SS, Agrawal-Singh S, Dietrich N, Christophersen NS, Helin K, Hansen K. 2010. Polycomb group protein displacement and gene activation through MSK-dependent H3K27me3S28 phosphorylation. Mol Cell 39: 886–900 20864036 10.1016/j.molcel.2010.08.020

[B17] Gehart H, Kumpf S, Ittner A, Ricci R. 2010. MAPK signalling in cellular metabolism: stress or wellness? EMBO Rep 11: 834–840 20930846 10.1038/embor.2010.160 PMC2966959

[B18] Gentleman RC, Carey VJ, Bates DM, Bolstad B, Dettling M, Dudoit S, Ellis B, Gautier L, Ge Y, Gentry J, . 2004. Bioconductor: open software development for computational biology and bioinformatics. Genome Biol 5: R80 15461798 10.1186/gb-2004-5-10-r80 PMC545600

[B19] Gilchrist DA, Dos Santos G, Fargo DC, Xie B, Gao Y, Li L, Adelman K. 2010. Pausing of RNA polymerase II disrupts DNA-specified nucleosome organization to enable precise gene regulation. Cell 143: 540–551 21074046 10.1016/j.cell.2010.10.004 PMC2991113

[B20] Glaser KB, Staver MJ, Waring JF, Stender J, Ulrich RG, Davidsen SK. 2003. Gene expression profiling of multiple histone deacetylase (HDAC) inhibitors: defining a common gene set produced by HDAC inhibition in T24 and MDA carcinoma cell lines. Mol Cancer Ther 2: 151–163 12589032

[B21] Guenther MG, Levine SS, Boyer LA, Jaenisch R, Young RA. 2007. A chromatin landmark and transcription initiation at most promoters in human cells. Cell 130: 77–88 17632057 10.1016/j.cell.2007.05.042 PMC3200295

[B22] Hahne F, Durinck S, Ivanek R, Mueller A, Lianoglou S. 2013. *Gviz: Plotting data and annotation information along genomic coordinates*. R package version 1.4.5

[B23] Halegoua S, Patrick J. 1980. Nerve growth factor mediates phosphorylation of specific proteins. Cell 22: 571–581 6256087 10.1016/0092-8674(80)90367-0

[B24] Hargreaves DC, Horng T, Medzhitov R. 2009. Control of inducible gene expression by signal-dependent transcriptional elongation. Cell 138: 129–145 19596240 10.1016/j.cell.2009.05.047 PMC2828818

[B25] Hauser C, Schuettengruber B, Bartl S, Lagger G, Seiser C. 2002. Activation of the mouse histone deacetylase 1 gene by cooperative histone phosphorylation and acetylation. Mol Cell Biol 22: 7820–7830 12391151 10.1128/MCB.22.22.7820-7830.2002 PMC134744

[B26] He S, Khan DH, Winter S, Seiser C, Davie JR. 2013. Dynamic distribution of HDAC1 and HDAC2 during mitosis: association with F-actin. J Cell Physiol 228: 1525–1535 23280436 10.1002/jcp.24311

[B27] Heinz S, Benner C, Spann N, Bertolino E, Lin YC, Laslo P, Cheng JX, Murre C, Singh H, Glass CK. 2010. Simple combinations of lineage-determining transcription factors prime *cis*-regulatory elements required for macrophage and B cell identities. Mol Cell 38: 576–589 20513432 10.1016/j.molcel.2010.05.004 PMC2898526

[B28] Herschman HR. 1991. Primary response genes induced by growth factors and tumor promoters. Annu Rev Biochem 60: 281–319 1883198 10.1146/annurev.bi.60.070191.001433

[B29] Huang da W, Sherman BT, Lempicki RA. 2009. Systematic and integrative analysis of large gene lists using DAVID bioinformatics resources. Nat Protoc 4: 44–57 19131956 10.1038/nprot.2008.211

[B30] Imhof A, Yang XJ, Ogryzko VV, Nakatani Y, Wolffe AP, Ge H. 1997. Acetylation of general transcription factors by histone acetyltransferases. Curr Biol 7: 689–692 9285713 10.1016/s0960-9822(06)00296-x

[B31] Johnson DG, Dent SY. 2013. Chromatin: receiver and quarterback for cellular signals. Cell 152: 685–689 23375745 10.1016/j.cell.2013.01.017 PMC3644977

[B32] Khurana A, Dey CS. 2003. p38 MAPK interacts with actin and modulates filament assembly during skeletal muscle differentiation. Differentiation 71: 42–50 12558602 10.1046/j.1432-0436.2003.700604.x

[B33] Kocher T, Pichler P, Swart R, Mechtler K. 2012. Analysis of protein mixtures from whole-cell extracts by single-run nanoLC-MS/MS using ultralong gradients. Nat Protoc 7: 882–890 22498708 10.1038/nprot.2012.036

[B34] Kurdistani SK, Grunstein M. 2003. Histone acetylation and deacetylation in yeast. Nat Rev Mol Cell Biol 4: 276–284 12671650 10.1038/nrm1075

[B35] Lagger S, Meunier D, Mikula M, Brunmeir R, Schlederer M, Artaker M, Pusch O, Egger G, Hagelkruys A, Mikulits W, . 2010. Crucial function of histone deacetylase 1 for differentiation of teratomas in mice and humans. EMBO J 29: 3992–4007 20967026 10.1038/emboj.2010.264 PMC3020644

[B36] Langmead B, Schatz MC, Lin J, Pop M, Salzberg SL. 2009. Searching for SNPs with cloud computing. Genome Biol 10: R134 19930550 10.1186/gb-2009-10-11-r134 PMC3091327

[B37] Lau PN, Cheung P. 2011. Histone code pathway involving H3 S28 phosphorylation and K27 acetylation activates transcription and antagonizes polycomb silencing. Proc Natl Acad Sci 108: 2801–2806 21282660 10.1073/pnas.1012798108 PMC3041124

[B38] Mahadevan LC, Willis AC, Barratt MJ. 1991. Rapid histone H3 phosphorylation in response to growth factors, phorbol esters, okadaic acid, and protein synthesis inhibitors. Cell 65: 775–783 2040014 10.1016/0092-8674(91)90385-c

[B39] Mahadevan LC, Clayton AL, Hazzalin CA, Thomson S. 2004. Phosphorylation and acetylation of histone H3 at inducible genes: two controversies revisited. Novartis Found Symp 259: 102–111; discussion 111–104, 163–109 15171249

[B40] Min IM, Waterfall JJ, Core LJ, Munroe RJ, Schimenti J, Lis JT. 2011. Regulating RNA polymerase pausing and transcription elongation in embryonic stem cells. Genes Dev 25: 742–754 21460038 10.1101/gad.2005511 PMC3070936

[B41] Mohn F, Weber M, Rebhan M, Roloff TC, Richter J, Stadler MB, Bibel M, Schubeler D. 2008. Lineage-specific polycomb targets and de novo DNA methylation define restriction and potential of neuronal progenitors. Mol Cell 30: 755–766 18514006 10.1016/j.molcel.2008.05.007

[B42] Muse GW, Gilchrist DA, Nechaev S, Shah R, Parker JS, Grissom SF, Zeitlinger J, Adelman K. 2007. RNA polymerase is poised for activation across the genome. Nat Genet 39: 1507–1511 17994021 10.1038/ng.2007.21 PMC2365887

[B43] Nascimento EM, Cox CL, MacArthur S, Hussain S, Trotter M, Blanco S, Suraj M, Nichols J, Kubler B, Benitah SA, . 2011. The opposing transcriptional functions of Sin3a and c-Myc are required to maintain tissue homeostasis. Nat Cell Biol 13: 1395–1405 22101514 10.1038/ncb2385 PMC3242072

[B44] Nechaev S, Fargo DC, dos Santos G, Liu L, Gao Y, Adelman K. 2010. Global analysis of short RNAs reveals widespread promoter-proximal stalling and arrest of Pol II in *Drosophila*. Science 327: 335–338 20007866 10.1126/science.1181421 PMC3435875

[B45] Pruitt KD, Tatusova T, Maglott DR. 2007. NCBI reference sequences (RefSeq): a curated non-redundant sequence database of genomes, transcripts and proteins. Nucleic Acids Res 35: D61–D65 17130148 10.1093/nar/gkl842 PMC1716718

[B46] R Development Core Team. 2014. R: *A language and environment for statistical computing*. R Foundation for Statistical Computing, Vienna, Austria. http://www.R-project.org/

[B47] Rahl PB, Lin CY, Seila AC, Flynn RA, McCuine S, Burge CB, Sharp PA, Young RA. 2010. c-Myc regulates transcriptional pause release. Cell 141: 432–445 20434984 10.1016/j.cell.2010.03.030 PMC2864022

[B48] Ramirez-Carrozzi VR, Braas D, Bhatt DM, Cheng CS, Hong C, Doty KR, Black JC, Hoffmann A, Carey M, Smale ST. 2009. A unifying model for the selective regulation of inducible transcription by CpG islands and nucleosome remodeling. Cell 138: 114–128 19596239 10.1016/j.cell.2009.04.020 PMC2712736

[B49] Rasmussen EB, Lis JT. 1993. In vivo transcriptional pausing and cap formation on three *Drosophila* heat shock genes. Proc Natl Acad Sci 90: 7923–7927 8367444 10.1073/pnas.90.17.7923 PMC47259

[B50] Robinson MD, McCarthy DJ, Smyth GK. 2010. edgeR: a Bioconductor package for differential expression analysis of digital gene expression data. Bioinformatics 26: 139–140 19910308 10.1093/bioinformatics/btp616 PMC2796818

[B51] Saccani S, Pantano S, Natoli G. 2002. p38-Dependent marking of inflammatory genes for increased NF-κ B recruitment. Nat Immunol 3: 69–75 11743587 10.1038/ni748

[B52] Sawicka A, Seiser C. 2012. Histone H3 phosphorylation—a versatile chromatin modification for different occasions. Biochimie 94: 2193–2201 22564826 10.1016/j.biochi.2012.04.018 PMC3480636

[B53] Schones DE, Cui K, Cuddapah S, Roh TY, Barski A, Wang Z, Wei G, Zhao K. 2008. Dynamic regulation of nucleosome positioning in the human genome. Cell 132: 887–898 18329373 10.1016/j.cell.2008.02.022 PMC10894452

[B54] Schroder S, Herker E, Itzen F, He D, Thomas S, Gilchrist DA, Kaehlcke K, Cho S, Pollard KS, Capra JA, . 2013. Acetylation of RNA polymerase II regulates growth-factor-induced gene transcription in mammalian cells. Mol Cell 52: 314–324 24207025 10.1016/j.molcel.2013.10.009 PMC3936344

[B55] Simboeck E, Sawicka A, Zupkovitz G, Senese S, Winter S, Dequiedt F, Ogris E, Di Croce L, Chiocca S, Seiser C. 2010. A phosphorylation switch regulates the transcriptional activation of cell cycle regulator p21 by histone deacetylase inhibitors. J Biol Chem 285: 41062–41073 20952396 10.1074/jbc.M110.184481 PMC3003405

[B56] Smith E, Shilatifard A. 2010. The chromatin signaling pathway: diverse mechanisms of recruitment of histone-modifying enzymes and varied biological outcomes. Mol Cell 40: 689–701 21145479 10.1016/j.molcel.2010.11.031 PMC3037032

[B57] Soloaga A, Thomson S, Wiggin GR, Rampersaud N, Dyson MH, Hazzalin CA, Mahadevan LC, Arthur JS. 2003. MSK2 and MSK1 mediate the mitogen- and stress-induced phosphorylation of histone H3 and HMG-14. EMBO J 22: 2788–2797 12773393 10.1093/emboj/cdg273 PMC156769

[B58] Soutoglou E, Katrakili N, Talianidis I. 2000. Acetylation regulates transcription factor activity at multiple levels. Mol Cell 5: 745–751 10882110 10.1016/s1097-2765(00)80253-1

[B59] Spite M, Baba SP, Ahmed Y, Barski OA, Nijhawan K, Petrash JM, Bhatnagar A, Srivastava S. 2007. Substrate specificity and catalytic efficiency of aldo-keto reductases with phospholipid aldehydes. Biochem J 405: 95–105 17381426 10.1042/BJ20061743 PMC1925154

[B60] Tan Y, Rouse J, Zhang A, Cariati S, Cohen P, Comb MJ. 1996. FGF and stress regulate CREB and ATF-1 via a pathway involving p38 MAP kinase and MAPKAP kinase-2. EMBO J 15: 4629–4642 8887554 PMC452194

[B61] Thomson S, Clayton AL, Mahadevan LC. 2001. Independent dynamic regulation of histone phosphorylation and acetylation during immediate-early gene induction. Mol Cell 8: 1231–1241 11779499 10.1016/s1097-2765(01)00404-x

[B62] Titov DV, Gilman B, He QL, Bhat S, Low WK, Dang Y, Smeaton M, Demain AL, Miller PS, Kugel JF, . 2011. XPB, a subunit of TFIIH, is a target of the natural product triptolide. Nat Chem Biol 7: 182–188 21278739 10.1038/nchembio.522 PMC3622543

[B63] Trapnell C, Pachter L, Salzberg SL. 2009. TopHat: discovering splice junctions with RNA-seq. Bioinformatics 25: 1105–1111 19289445 10.1093/bioinformatics/btp120 PMC2672628

[B64] Weiner A, Chen HV, Liu CL, Rahat A, Klien A, Soares L, Gudipati M, Pfeffner J, Regev A, Buratowski S, . 2012. Systematic dissection of roles for chromatin regulators in a yeast stress response. PLoS Biol 10: e1001369 22912562 10.1371/journal.pbio.1001369 PMC3416867

[B65] Wiggin GR, Soloaga A, Foster JM, Murray-Tait V, Cohen P, Arthur JS. 2002. MSK1 and MSK2 are required for the mitogen- and stress-induced phosphorylation of CREB and ATF1 in fibroblasts. Mol Cell Biol 22: 2871–2881 11909979 10.1128/MCB.22.8.2871-2881.2002 PMC133730

[B66] Winter S, Fischle W, Seiser C. 2008a. Modulation of 14-3-3 interaction with phosphorylated histone H3 by combinatorial modification patterns. Cell Cycle 7: 1336–1342 18418070 10.4161/cc.7.10.5946 PMC3182529

[B67] Winter S, Simboeck E, Fischle W, Zupkovitz G, Dohnal I, Mechtler K, Ammerer G, Seiser C. 2008b. 14-3-3 proteins recognize a histone code at histone H3 and are required for transcriptional activation. EMBO J 27: 88–99 18059471 10.1038/sj.emboj.7601954 PMC2206135

[B68] Yamamoto Y, Verma UN, Prajapati S, Kwak YT, Gaynor RB. 2003. Histone H3 phosphorylation by IKK-α is critical for cytokine-induced gene expression. Nature 423: 655–659 12789342 10.1038/nature01576

[B69] Yang C, Bolotin E, Jiang T, Sladek FM, Martinez E. 2007. Prevalence of the initiator over the TATA box in human and yeast genes and identification of DNA motifs enriched in human TATA-less core promoters. Gene 389: 52–65 17123746 10.1016/j.gene.2006.09.029 PMC1955227

[B70] Zeitlinger J, Stark A, Kellis M, Hong JW, Nechaev S, Adelman K, Levine M, Young RA. 2007. RNA polymerase stalling at developmental control genes in the *Drosophila melanogaster* embryo. Nat Genet 39: 1512–1516 17994019 10.1038/ng.2007.26 PMC2824921

[B71] Zhang Y, Liu T, Meyer CA, Eeckhoute J, Johnson DS, Bernstein BE, Nusbaum C, Myers RM, Brown M, Li W, . 2008. Model-based analysis of ChIP-seq (MACS). Genome Biol 9: R137 18798982 10.1186/gb-2008-9-9-r137 PMC2592715

[B72] Zhu LJ, Gazin C, Lawson ND, Pages H, Lin SM, Lapointe DS, Green MR. 2010. ChIPpeakAnno: a bioconductor package to annotate ChIP-seq and ChIP-chip data. BMC Bioinformatics 11: 237 20459804 10.1186/1471-2105-11-237 PMC3098059

[B73] Zippo A, Serafini R, Rocchigiani M, Pennacchini S, Krepelova A, Oliviero S. 2009. Histone crosstalk between H3S10ph and H4K16ac generates a histone code that mediates transcription elongation. Cell 138: 1122–1136 19766566 10.1016/j.cell.2009.07.031

